# Effects of Variations in Resistance Training Frequency on Strength Development in Well-Trained Populations and Implications for In-Season Athlete Training: A Systematic Review and Meta-analysis

**DOI:** 10.1007/s40279-021-01460-7

**Published:** 2021-04-22

**Authors:** Matthew Cuthbert, G. Gregory Haff, Shawn M. Arent, Nicholas Ripley, John J. McMahon, Martin Evans, Paul Comfort

**Affiliations:** 1 Human Performance Laboratory, Directorate of Sport, Exercise, and Physiotherapy, University of Salford, Greater Manchester, UK; 2 Technical Directorate Division, The FA Group, St George’s Park, Burton-Upon-Trent, Staffordshire, UK; 3 School of Medical and Health Sciences, School of Exercise and Health Sciences, Edith Cowan University, Joondalup, Australia; 4 Department of Exercise Science, Arnold School of Public Health, University of South Carolina, Columbia, South Carolina USA; 5 Institute for Sport, Physical Activity and Leisure, Carnegie School of Sport, Leeds Beckett University, Leeds, UK

## Abstract

**Background:**

In-season competition and tournaments for team sports can be both long and congested, with some sports competing up to three times per week. During these periods of time, athletes need to prepare technically, tactically and physically for the next fixture and the short duration between fixtures means that, in some cases, physical preparation ceases, or training focus moves to recovery as opposed to progressing adaptations.

**Objective:**

The aim of this review was to investigate the effect of training frequency on muscular strength to determine if a potential method to accommodate in-season resistance training, during busy training schedules, could be achieved by utilizing shorter more frequent training sessions across a training week.

**Methods:**

A literature search was conducted using the SPORTDiscus, Ovid, PubMed and Scopus databases. 2134 studies were identified prior to application of the following inclusion criteria: (1) maximal strength was assessed, (2) a minimum of two different training frequency groups were included, (3) participants were well trained, and finally (4) compound exercises were included within the training programmes. A Cochrane risk of bias assessment was applied to studies that performed randomized controlled trials and consistency of studies was analysed using *I*^2^ as a test of heterogeneity. Secondary analysis of studies included Hedges’ g effect sizes (*g*) and between-study differences were estimated using a random-effects model.

**Results:**

Inconsistency of effects between pre- and post-intervention was low within-group (*I*^2^ = 0%), and moderate between-group (*I*^2^ ≤ 73.95%). Risk of bias was also low based upon the Cochrane risk of bias assessment. Significant increases were observed overall for both upper (*p* ≤ 0.022) and lower (*p* ≤ 0.008) body strength, pre- to post-intervention, when all frequencies were assessed. A small effect was observed between training frequencies for upper (*g* ≤ 0.58) and lower body (*g* ≤ 0.45).

**Conclusion:**

Over a 6–12-week period, there are no clear differences in maximal strength development between training frequencies, in well-trained populations. Such observations may permit the potential for training to be manipulated around competition schedules and volume to be distributed across shorter, but more frequent training sessions within a micro-cycle rather than being condensed into 1–2 sessions per week, in effect, allowing for a micro-dosing of the strength stimuli.

**Supplementary Information:**

The online version contains supplementary material available at 10.1007/s40279-021-01460-7.

## Key Points

**Table:** 

Muscular strength is an integral component of sporting demands, with athletes required to repeatedly exert a large magnitude of force on external objects.
A number of team sports have long seasons and short off/pre-seasons, whereby making improvements in muscular strength whilst also managing fatigue can become conflicting.
The vast majority of interventions comparing resistance-training frequencies have used a moderate load, high volume threshold of 8–12 repetition maximum, which may not optimally increase strength in-season.
There appears to be no clear difference between resistance-training frequencies when volume is equated, suggesting potential flexibility in resistance-training prescription across a micro-cycle.

## Background

The basic demands of many sports require athletes to rapidly exert high forces to accelerate or decelerate external objects [1, 2] and/or manipulate their own body mass, an opponent’s mass in addition to their own, or an implement and/or projectile [1]. Resistance training focused primarily on the development of strength, rather than strength endurance or hypertrophy, is arguably the most critical focus for improving athletic performance and underpins both individual and team sports [1, 3]. Evidence of this has been provided through numerous studies demonstrating moderate-to-large correlations between maximal strength and dynamic performance [1]. In addition, strength training has also been outlined as a potent method to reduce the risk of muscular injuries [4, 5] with well-developed lower body strength, repeated sprint ability, and speed increasing an athlete’s tolerance to higher training loads and in turn reducing risk of injury compared to lower performance groups in the aforementioned areas [6].

Resistance training (RT) has long been used to improve skeletal muscle function, architecture and activation. The manipulation of sets, repetitions, and load lifted (usually as a percentage of repetition maximum [RM]) during RT dictates the muscular and neurological adaptations. Typically, RT application consists of a focus on strength (low volume [3–5 sets of ≤ 6 repetitions]—high load [≥ 85% 1RM]), hypertrophy (high volume [3–5 sets of 6–12 repetitions]—moderate load [67–85% 1RM]), endurance (high volume [~ 3 sets of ≥ 12 repetitions]—low load [≤ 67% 1RM]) [7], or power. Increased power output can be achieved via numerous means; for example, in untrained/weak individuals this can be accomplished via increased focus on basic strength training [8, 9]. Stronger athletes, however, need to include loaded high velocity ballistic tasks such as weightlifting [1, 2] and higher velocity jump and plyometric training, using minimal additional load [8, 10], or depending upon the periodization model used, a mixture of each of these modes (strength, ballistic and plyometric training) can be employed [11]. Within periods of an athlete’s career, across each season, and based on the athlete’s training status and goals, the emphasis on certain training foci is more appropriate than others. Appropriately planned RT can help to increase specific musculoskeletal and neurological adaptations through the manipulation of and interaction between specific training principles, including volume (sets x repetitions), load (often referred to as intensity) and frequency.

The current American College of Sports Medicine (ACSM) guidelines suggest that, for ‘general muscular fitness’ training, sessions should occur 2–3 days per week with 48 h recovery in between [12]. The National Strength and Conditioning Association (NSCA) also suggests training frequencies of 2–3 times per week for beginners, 3–4 times per week for intermediates and 4–7 times per week for those with advanced training status [7]. Within an athletic population, however, such training frequencies are generally unrealistic due to the demands of in-season competition, especially within team sports where competitions can be as frequent as 2–3 times per week. In well-trained/athletic populations, RT typically follows some form of periodization. The traditional approach to periodization, whereby the competition year/macrocycle is divided into a preparation, competition and transition period, as seen in the model outlined by Matveyev [13], was originally developed for individual athletes where ‘maintenance’ of strength is the primary goal during a relatively short competition period. Maintenance of strength and athletic abilities during the competition period is, therefore, not always appropriate within all team sport settings, due to their competition period lasting between 3 and 9 months, depending on the sport. Effectiveness and duration of off-season/pre-season preparatory periods, for the development of physical qualities, need to be considered when planning and implementing in-season training priorities. Density of competition does, however, play a role in the planning and regularity of RT with basketball teams required to play three times per week, and some soccer teams required to compete three times in 10 days. International tournaments also include compact fixture schedules, where depending upon the age group competing, games are typically played 72 h apart [14] but can be played between 48 and 96 h apart. The ability to provide sufficient periods of recovery, as well as opportunities to provide stimuli for the maintenance of strength through RT, become limited during these periods of congested fixtures when following the ACSM frequency guidelines [12].

Lundberg et al. [15] concluded that 2 resting days (~ 72 h between matches) are not sufficient for players to recover from match-induced muscle soreness during congested periods. Although the NSCA guidelines do take fixtures into account by recommending a reduced RT frequency of 1–3 times per week in-season compared to their usual recommendations [7], this is not entirely dissimilar from the ACSM’s recommendation of 2–3 times a week and, therefore, the same argument could apply. Athletes with greater strength, high-intensity running capability and aerobic capacity have demonstrated an ability to recover quicker than those less conditioned, despite having worked at higher intensities during competition [16, 17]. Greater ability to recover may allow for the two rest days between fixtures, highlighted by Lundberg et al. [15] to allow sufficient recovery; however this has not been investigated over a period of chronic fixture congestion. In-season strength maintenance has only been exhibited over a 12-week period, whereby Rønnestad et al. [18] demonstrated that RT once per week, over the first 12 weeks of the season, permitted the maintenance of strength, compared to one session every two weeks which resulted in an average strength loss of 10% in a 1RM half squat. For sports where there are regularly ≥ 2 games per week, and a large technical-tactical focused approach, accompanied by a low-intensity recovery focus [19], there would potentially be a detrimental long-term effect as the athletes would not be able to maintain strength throughout a full season. The majority of team sports’ competitive seasons last longer than 12 weeks, however, suggesting there needs to be a greater level of insight into whether the lower end of the NSCA’s frequency guidelines are appropriate for competition periods > 12 weeks. The ‘maintenance’ of strength throughout a season/tournament is, therefore, likely to be most appropriate during short, condensed seasons, as seen in many U.S. collegiate sports [18] whereby the teams that decline the least over that short period will likely improve their chances of success. These athletes also benefit from having relatively long periods to enhance their physical capabilities prior to and following competition. Focusing on ‘maintenance’ is arguably a poor training goal during long seasons, much like those seen in the National Basketball Association, National Hockey League and soccer leagues worldwide, especially in light of the fact that small but progressive increases in performance can be achieved depending on training status of the athletes and periodization model used [20]. When the importance of competition increases as the competitive season progresses, with teams competing in play-offs or knockout stages of competitions, performance needs to be high and injuries minimized during these latter stages of competition. With limited off/pre-seasons, it is, therefore, important to continue strength development during the season to ensure the best athletes are available and appropriately prepared.

Resistance training frequency has previously been reviewed meta-analytically [21, 22], with what appears to be differing conclusions regarding the improvements in strength through RT. Grgic et al. [21] attributed increased RT frequency to increased ‘gains’ in muscular strength, whereas Ralston et al. [22] report no significant differences (*p* = 0.25) between low- and high-frequency training. Grgic et al. [21] are explicit, however, in describing that when their data set is analysed in sub-groups, no significant differences (*p* = 0.324) occurred between groups when volume was equated, suggesting that weekly RT volume was the underlying determinant of improvements in muscular strength, rather than training frequency. The limitations of both reviews are due to the populations included, both having analysed data that included largely untrained populations and young, middle-aged, and older subjects. Evidence provided through the use of untrained populations is not always valid when making comparisons with trained individuals and athletic populations due to untrained populations responding and adapting favorably to a multitude of different stimuli [23]. Inspection of the appropriateness of exercise prescription within the interventions was not included in either of the aforementioned meta-analyses, with the authors only critiquing the lack of equated volumes in some cases, whereas most of the studies included did not prescribe repetitions, sets and load based upon recommended strength training thresholds (3–5 sets, ≤ 6 repetitions, load ≥ 85% 1RM) [7] but rather those more appropriate or ‘optimal’ for hypertrophy (2–5 sets, 8–12 repetitions, loads equivalent to ≤ 80% 1RM) [7, 21, 22].

The aim of this systematic review and meta-analysis was to assess the effect of RT frequency on maximum strength in athletic and well-trained populations to provide possible implications for how this may affect practitioners’ in-season RT prescription.

## Methods

### Study design

This systematic review design was developed in adherence to the guidelines of the Preferred Reporting Items for Systematic Reviews and Meta-analysis (PRISMA). The PRISMA guidelines include 27 items within a checklist that is designed to be used as the basis for reporting systematic reviews [24]. The research question being investigated within this review as defined by the PICO model (population, intervention, comparison and outcome) is whether in a well-trained population, during volume matched resistance-training interventions, does the frequency of training have an effect on both lower and upper body strength outcomes during experimental randomized and non-randomized studies? A protocol was not pre-registered for this review.

### Literature Search

A Boolean/phrase search mode was applied using the following keywords: “resistance training” AND “frequency” AND “volume” AND “intensity”. The keywords were inputted using this format into four different databases, including PubMed, SPORTDiscus, Ovid and Scopus. Filters were applied to all databases to include studies that were written in the English language and presented in peer-reviewed academic journal articles. No restrictions were placed upon the sex of subjects; age, however, was restricted to no greater than 35 years, with no lower age cut-off.

### Inclusion and Exclusion Criteria

**Fig. 1:**
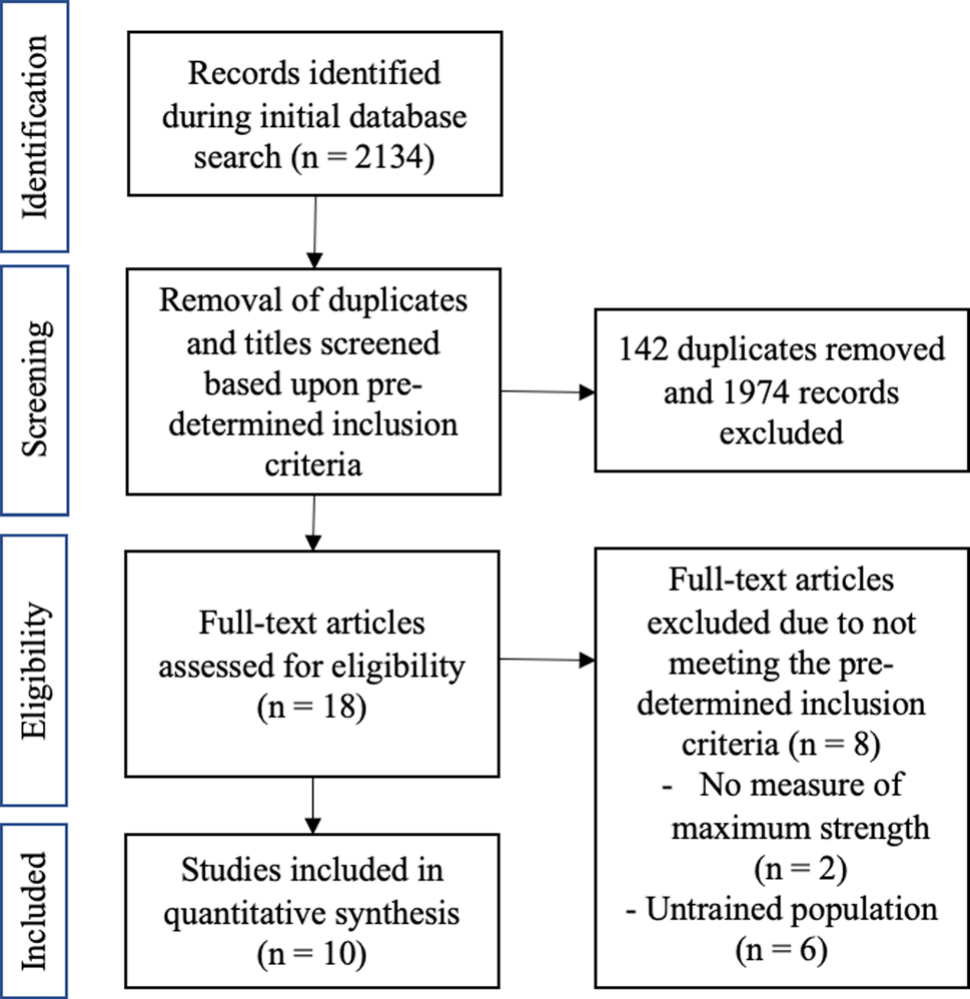
PRISMA flowchart

The primary focus of this literature search was to identify studies that have assessed the effect of different RT frequencies in trained/athletic populations. The search timeframe was restricted up to and including 1^st^ April 2020, with no earliest date restriction, and following this search there were 2134 articles identified for further inspection. All duplicated studies were removed initially with the remaining studies then being screened utilizing the subsequent criteria. Research articles were included and eligible within this review provided that (1) a measure of maximal strength was assessed, (2) a minimum of two different training frequency groups were included, (3) the populations of the studies were stated as well trained, and finally (4) multi-joint exercises were included within the training programmes. Studies were excluded for using subjects that were not injury free for the 6 months prior, along with any systematic or narrative reviews. A summary of the above selection process is outlined in Fig. 1. Means and standard deviation (SD) were required from all papers to be analysed further; if these values were not present but the study met the rest of the criteria, the corresponding authors were contacted in order to obtain these values.

### Quality and Risk of Bias Assessment

Following the identification of the studies included within this review, the quality and risk of bias were assessed. This included a Cochrane risk of bias assessment tool to assess the risk of bias within the randomized controlled trials. The Cochrane risk of bias assessment tool evaluates randomized controlled trials based on several categories that include sequence generation, allocation concealment, blinding of participants and personnel, blinding of outcome assessment, incomplete outcome data, selective outcome reporting, and ‘other issues.’ Grades for these categories were provided as either ‘high risk of bias’, ‘low risk of bias’, or ‘unclear risk of bias’.

### Analysis and Interpretation of Results

Means and SDs of upper and lower body maximal strength measures were independently extracted from the included studies for further analysis. Maximal strength tests included 1RM back squat, leg press, and bench press, and maximum voluntary isometric contraction of the knee and elbow flexors. Hedge’s g effect sizes (*g*) were calculated from the pre- to post-intervention results of each study to provide a standardized value whereby the magnitude of differences can be determined and compared across interventions whilst accounting for differences in sample size. The scale for interpretation of *g* was proposed by Hopkins [25] as follows: trivial (≤ 0.20), small (0.21–0.59), moderate (0.60–1.19), large (1.20–1.99), or very large (≥ 2.00). An estimation for between-study variance was calculated using a random-effects model, with associated Z value, *p* value and 95% confidence intervals (95% CI), absolute heterogeneity was assessed using Tau^2^ and this was estimated using the restricted maximum likelihood method. Finally, a test for relative heterogeneity (*I*^*2*^), as outlined by Higgins et al. [26], was used to quantify the relative inconsistency of effects, using a scale of low (< 25%), moderate (25–75%) and high (≥ 75%) and the associated significance with an a priori alpha level of *p* < 0.05. All statistical analyses were carried out using Jamovi [27]. Training frequency of the lower and upper body was defined as the number of sessions that included exercises targeting those areas, respectively, per week. Comparisons were made between the lower frequency and higher frequency groups within each study. Training frequencies can be interpreted across the whole spectrum, with some sports considering a ‘high’ frequency to be the equivalent of a ‘low’ frequency in other sports; for example, within basketball, the majority of the season is spent playing three games per week, whereby one or two dedicated RT sessions would be considered high frequency, compared to a sport such as rugby or American Football whereby that same frequency would be considered low. From a research perspective, there is also no clear definition of what constitutes high and low frequencies, with some studies for example labeling three sessions a week as low [28, 29] frequency and some as high [30–33]. The authors, therefore, have not definitively classified any frequency as either being low or high but made comparisons as lower and higher. Due to body mass not being reported for individual groups in all of the included studies both pre- and post-intervention, changes in relative strength of each group could not be assessed. Instead, baseline strength between groups within each study were compared separately to highlight magnitude of strength differences between the study groups. All studies included within the meta-analyses were independently evaluated by two of the authors (NR and PC) for methodological quality.

## Results

### Search Results

Two thousand, one hundred and thirty-four studies were identified within the four databases highlighted in Sect. 2.2. Figure 1 illustrates that of the total studies identified, 142 articles were duplicates and, therefore, removed first. Following the application of the predetermined inclusion/exclusion criteria to both titles and abstracts of the identified studies, and with further inspection of the full text if required, a total of ten studies remained for further analysis [28–37].

### Systematic Review and Meta-analyses Findings

The results of two different meta-analyses were calculated to evaluate the effectiveness of the interventions on both lower and upper body strength pre- and post-intervention as well as the differences in effect between the lower and higher frequencies for each study (Table 1). Pre- to post-intervention *g* values can be seen in Supplementary Information Figures S1 and S2, with most of the interventions, regardless of frequency, demonstrating small-to-moderate (*g* = − 0.40–1.11) increases in strength. The estimated overall effect for strength in both the lower body (*g* = 0.562) and upper body (*g* = 0.323) pre- to post-intervention demonstrated the effectiveness of resistance training with significant increases for each of the meta-analyses, respectively (*p* < 0.001) (Table 1). When comparing lower and higher frequencies groups of each study, no overall significant effect was observed for lower body (*p* = 0.453 and *g* = 0.088) and upper body (*p* = 0.505 and *g* = 0.088).

**Table 1:** A summary of the meta-analytical statistics for intervention effect and frequency differences

	Overall effect	Z	*p*	95% CI	Tau^2^	I^2^ (%)	*p*
*Pre- vs post-intervention*							
Upper body	0.323	3.674	< 0.001	0.151–0.495	< 0.001	0.00	0.983
Lower body	0.562	6.309	< 0.001	0.387–0.737	< 0.001	0.00	0.881
*Lower vs higher frequency*							
Upper body	0.088	0.667	0.505	− 0.171–0.348	< 0.001	0.00	0.990
Lower body	0.061	0.453	0.651	− 0.202–0.323	< 0.001	0.00	0.851

*Z* z score, *CI* confidence interval

### Study Quality and Bias Results

**Fig. 2:**
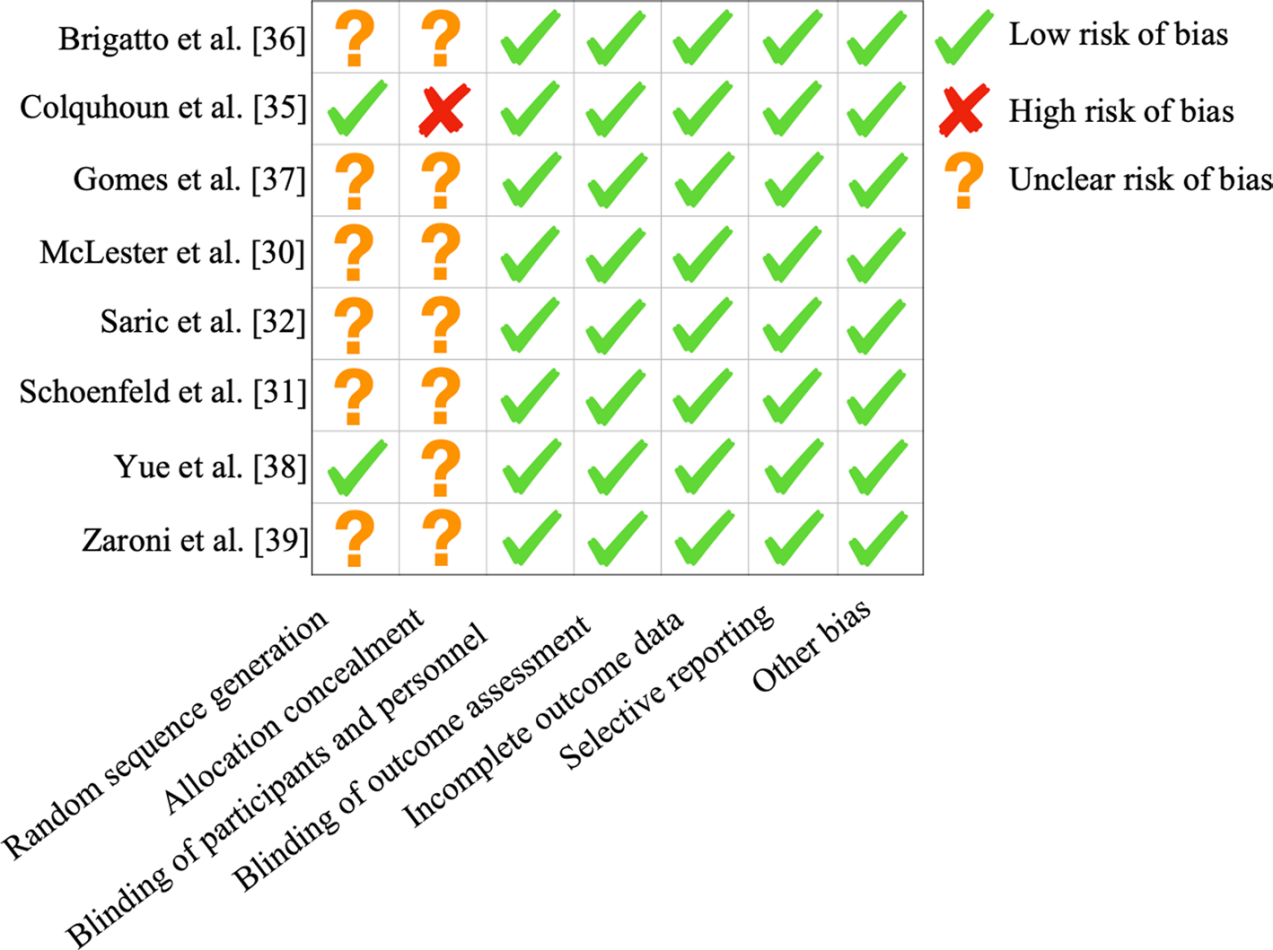
Depiction of the Cochrane risk of bias assessment

Heterogeneity assessments of the completed meta-analyses were conducted and can be seen in Table 1, with inconsistency of effects being extremely low pre- to post-intervention (Tau^2^ =  < 0.001, *I*^2^ = 0%). A Cochrane risk of bias assessment was completed (Fig. 2) on all studies that described some level of randomization within their methods, with the results of this generally showing low risk of bias around the reporting of blinding of participants as this was unlikely to influence the results. Selection bias for the most part was unclear as the majority of the studies, although stating that groups were randomly allocated, did not report methods of allocation.

## Discussion

The purpose of this review was to identify the effect of different RT training frequencies on maximum strength in well-trained populations to understand the influence different RT frequencies may have on athletes’ strength levels within-season. The findings of the systematic review and meta-analyses demonstrate that although the majority of interventions demonstrated significant and small positive effects (*p* < 0.001 and *g* ≤ 0.562) of resistance training (pre- to post-intervention) for upper (*p* = 0.505 and *g* = 0.088) and lower body (*p* = 0.651 and *g* = 0.061) strength, there was no significant difference and trivial effect in regard to the frequency of training, when volume was equated (Table 1). A Cochrane risk of bias assessment was used to assess the quality and bias of the studies reviewed, the results of which showed low risk of bias for the majority of categories assessed, with unclear bias around allocation concealment and method of random sequence generation. The findings of this review, therefore, agree with previous meta-analyses conducted by both Grgic et al. [21] and Ralston et al. [22] who investigated training frequency within a number of different, mostly untrained, populations suggesting that RT frequency has no significant effect on strength when volume is equated.

### Intervention Frequency

**Fig. 3:**
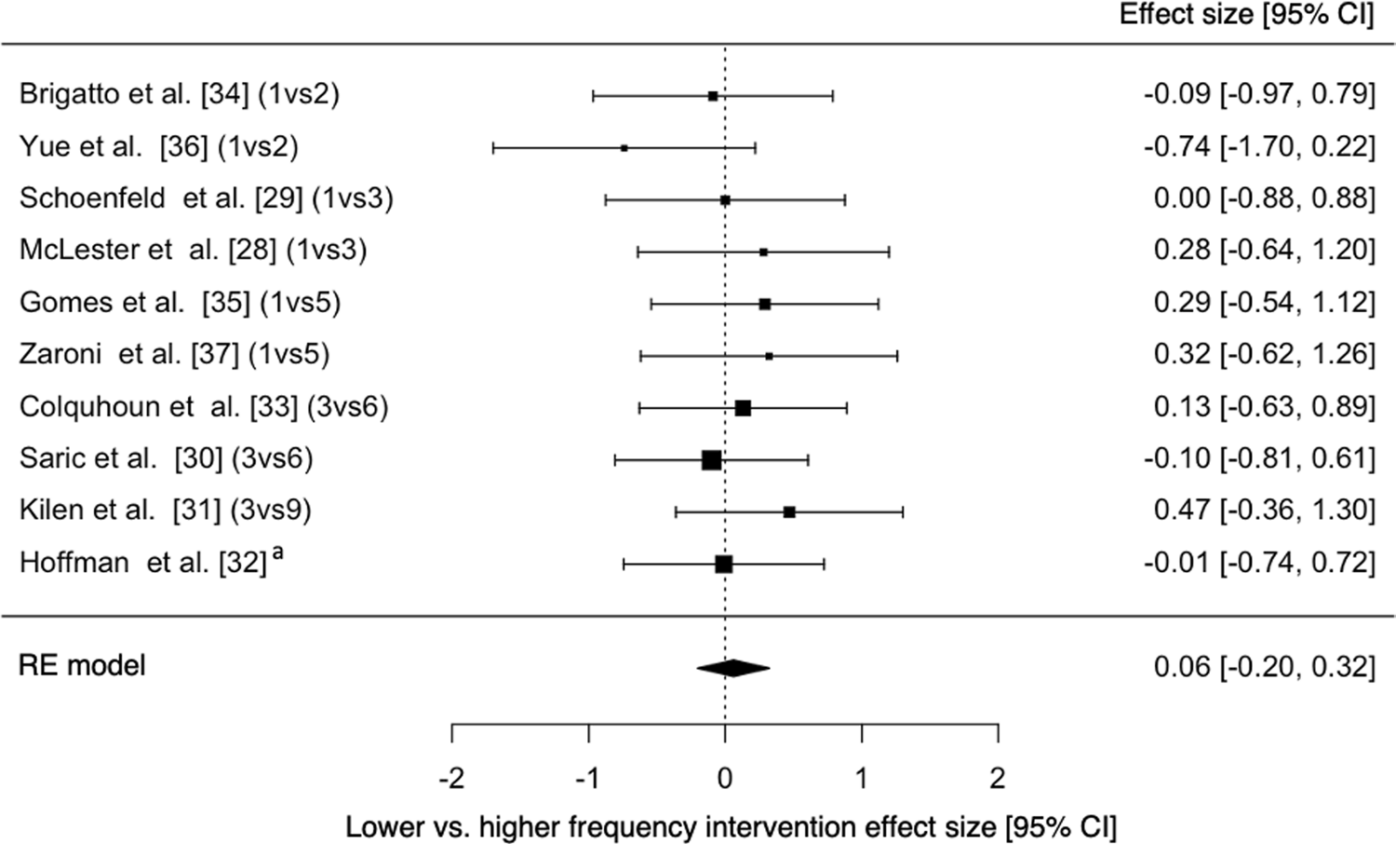
Differences in effect size between lower frequency and higher frequency groups on lower body strength (positive values favor the higher frequency groups and negative values favor the lower frequency groups). (1vs2) = once-weekly vs twice-weekly, (1vs3) = once-weekly vs 3 x/week, (1vs5) = once-weekly vs 5 x/week, (3vs6) = 3 x/week vs 6 x/week, (3vs9) = 3 x/week vs 9 x/week. ^a^ Aggregation of effect sizes due to the study comparing more than two groups. RE = random effects, CI = confidence interval

Due to a whole range of frequencies being investigated across the studies analysed in this review (Tables 2 and 3), the authors were unable to make enough comparisons to moderate the meta-analyses based on the frequency of each group. The lack of grouping has, therefore, led to some crossover between studies that have used one frequency as the ‘higher’ frequency that has also been included in a different study as the ‘lower’ frequency. An example of the crossover in training frequencies is demonstrated by McLester et al. [28] and Schoenfeld et al. [29] who both utilized three times per week as their higher RT frequency, whereas three times per week was used as the lower frequency in a number of the other studies included [30–33]. Although the crossover between descriptors of ‘lower’ and ‘higher’ frequencies may appear to be a possible issue in the reporting of data, it is important to bear in mind that the differences in effect observed range from trivial to small (g = − 0.10–0.33) for both lower and upper body regardless of the descriptor used. It is possible, however, to make a number of direct comparisons based on studies that utilized the same frequencies within their interventions. As mentioned previously, McLester et al. [28] and Schoenfeld et al. [29] both investigated once per week compared to three times per week, with neither resulting in once a week being favorable compared to three times per week. When comparing three times a week as a lower frequency, in studies conducted by Colquhoun et al. [33] and Saric et al. [30] who investigated three times per week compared to six times per week, the results were mixed. Lower body strength improved to a greater extent for the group training three times per week in the study by Saric et al. [30], whereas the greater improvement in the study by Colquhoun et al. [33] was observed in the six times per week group. The reverse was true for the upper body. The only frequency analysed within this review that consistently demonstrated superior effect when compared with others for lower body strength was five times per week. Gomes et al. [35] and Zaroni et al. [37] both investigated once per week compared to five times per week, with both favoring the higher frequency for lower body strength. A similar trend was shown by Gomes et al. [35] in the upper body; however, Zaroni et al. [37] found that once a week demonstrated greater increases in upper body strength. Although the study by Hoffman et al. [32] observed in Fig. 3 very slightly favors ‘lower’ frequency, this is likely due to the study itself investigating four different frequencies and, therefore, the results were aggregated; when comparing all the groups individually the trend observed by Gomes et al. [35] and Zaroni et al. [37] is also demonstrated, with five times per week consistently demonstrating the greater effect for the lower body (Supplementary Information Fig. S3). When inspecting the upper body strength changes individually for the study by Hoffman et al. [32], the pattern followed the same trend as the study by Saric et al. [30] whereby six times per week was consistently the superior frequency (Supplementary Information Fig. S4). Again, however, the differences in observed effect between the studies was trivial to small, much like the overall effect of the meta-analyses which also demonstrated non-significant differences. Due to the low heterogeneity for both the upper and lower body observed in Table 1 (*I*^*2*^ = 0%), there would continue to be minimal differences even if the sampling error of the interventions were to be removed.

**Table 2:** Characteristics of the training frequency interventions used for the lower body in the studies included within this review

Study	Title	Subjects	Population training status	Duration	Strength measures	Volume matched	Set x rep ranges	Frequencies (x a week)	Relative strength at baseline	Subject age (years)
Brigatto et al. [34]	Effect of resistance training frequency on neuromuscular performance and muscle morphology after eight weeks in trained men	n = 20	RT experience 4.1 ± 1.8 years	8 weeks	1RM Back squat	Yes	8 × 8-12RM	1	1.59 (kg.kg^−1^)	﻿27.1 ± 5.5
								2	1.61 (kg.kg^−1^)	
Colquhoun et al. [33]	Training volume, not frequency, indicative of maximal strength adaptations to resistance training	n = 28	RT training minimum of 3 × a week for 6 months and 150% of BW for deadlift 1RM	6 weeks	1RM Back squat	Yes	4 × 3–8 (Daily undulating)	3	1.73 (kg.kg^−1^)	﻿22 ± 2
								6	1.65 (kg.kg^−1^)	22 ± 3
Gomes et al. [35]	High-frequency resistance training is not more effective than low-frequency resistance training in increasing muscle mass and strength in well-trained men	n = 23	RT Experience 6.9 ± 3.1 years	8 weeks	1RM Back squat	Yes	10 × 8-12RM	1	1.70 (kg.kg^−1^)	﻿25.5 (24.0 – 26.5)
								5	1.56 (kg.kg^−1^)	27.1 (25.0 – 28.7)
Hoffman et al. [32]	The effects of self-selection for frequency of training in a winter conditioning program for football	n = 61	NCAA Division I athletes	10 weeks	1RM Back squat	No	5 × 2–10	2	1.84 (kg.kg^−1^)	20.1 ± 1.5
								3	1.74 (kg.kg^−1^)	19.7 ± 1.4
								5	1.72 (kg.kg^−1^)	20.1 ± 1.1
								6	1.71 (kg.kg^−1^)	19.7 ± 1.1
Kilen et al. [31]	Adaptations to short, frequent sessions of endurance and strength training are similar to longer, less frequent exercise sessions when the total volume is the same	n = 29	Military personnel with a minimum of 6 months RT experience	8 weeks	MVIC Knee extensor	No	2–3 × 8RM Lower body 2–3 × 5RM upper body	3	8.99 (N.kg^−1^)	22 ± 3
								9	9.06 (N.kg^−1^)	25 ± 3
McLester et al. [28]	Comparison of 1 day and 3 days per week of equal-volume resistance training in experienced subjects	n = 25	Minimum of 12 weeks RT experience	12 weeks	1RM Leg press	Yes	3 × 3—10 Lower body 3 × 5—10 upper body (muscle failure)	1	2.60 (kg.kg^−1^)	﻿26.0 ± 3.8
								3	2.65 (kg.kg^−1^)	﻿23.8 ± 5.4
Saric et al. [30]	Resistance training frequencies 3- and 6-times per week produce similar muscular adaptations in resistance-trained men	n = 27	RT training minimum of 2 × a week for 6 months	6 weeks	1RM Back squat	Yes	4 × 6-12RM (Muscle failure)	3	1.41 (kg.kg^−1^)	﻿22.6 ± 2.1
								6		
Schoenfeld et al. [29]	Influence of resistance training frequency on muscle adaptations in well-trained men	n = 20	RT training minimum of 3 × a week for 1 year	8 weeks	1RM Back squat	Yes	2–3 × 8–12 (Muscle failure)	1 (Split)	1.47 (kg.kg^−1^)	﻿23.5 ± 2.9
								3 (Total)		
Yue et al. [36]	Comparison of two equated resistance training weekly volume routines using different frequencies on body composition and performance in trained males	n = 18	RT Experience 3.0 ± 0.5 years	6 weeks	1RM Back squat	Yes	4 × 8–12	1	1.17 (kg.kg^−1^)	﻿﻿28 ± 7.9
								2	1.3 (kg.kg^−1^)	21 ± 3.2
Zaroni et al. [37]	High resistance-training frequency enhances muscle thickness in resistance-trained men	n = 18	RT experience range from 2–10 years	8 weeks	1RM Back squat	Yes	3 × 10–12	1 (Split)	1.33 (kg.kg^−1^)	﻿26.4 ± 4.6
								5 (Total)		

*RT* resistance training, *RM* repetition maximum, *BW* bodyweight, *DOMS* delayed onset muscle soreness, *MVIC* maximum voluntary isometric contraction

**Table 3:** Characteristics of the training frequency interventions used for the upper body in the studies included within this review

Study	Title	Subjects	Population training status	Duration	Strength measures	Volume matched	Set x rep ranges	Frequencies (x a week)	Relative strength at baseline	Subject age (years)
Brigatto et al. [34]	Effect of resistance training frequency on neuromuscular performance and muscle morphology after eight weeks in trained men	n = 20	RT experience 4.1 ± 1.8 years	8 weeks	1RM Bench press	Yes	8 × 8-12RM	1	1.19 (kg.kg^−1^)	﻿27.1 ± 5.5
								2	1.23 (kg.kg^−1^)	
Colquhoun et al. [33]	Training volume, not frequency, indicative of maximal strength adaptations to resistance training	n = 28	RT training minimum of 3 × a week for 6 months and 150% of BW for deadlift 1RM	6 weeks	1RM Bench press	Yes	4 × 3–8 (Daily undulating)	3	1.28 (kg.kg^−1^)	﻿22 ± 2
								6	1.22 (kg.kg^−1^)	22 ± 3
Gomes et al. [35]	High-frequency resistance training is not more effective than low-frequency resistance training in increasing muscle mass and strength in well-trained men	n = 23	RT Experience 6.9 ± 3.1 years	8 weeks	1RM Bench press	Yes	10 × 8-12RM	1	1.32 (kg.kg^−1^)	﻿25.5 (24.0 – 26.5)
								5	1.28 (kg.kg^−1^)	27.1 (25.0 – 28.7)
Hoffman et al. [32]	The effects of self-selection for frequency of training in a winter conditioning program for football	n = 61	NCAA Division I athletes	10 weeks	1RM Bench press	No	5 × 2–10	3	1.33 (kg.kg^−1^)	19.7 ± 1.4
								4	1.36 (kg.kg^−1^)	20.1 ± 1.5
								5	1.32 (kg.kg^−1^)	20.1 ± 1.1
								6	1.28 (kg.kg^−1^)	19.7 ± 1.1
Kilen et al. [31]	Adaptations to short, frequent sessions of endurance and strength training are similar to longer, less frequent exercise sessions when the total volume is the same	n = 29	Military personnel with a minimum of 6 months RT experience	8 weeks	MVIC Elbow flexor	No	2–3 × 8RM Lower body 2–3 × 5RM upper body	3	4.73 (N.kg^−1^)	22 ± 3
								9	5.51 (N.kg^−1^)	25 ± 3
McLester et al. [28]	Comparison of 1 day and 3 days per week of equal-volume resistance training in experienced subjects	n = 25	Minimum of 12 weeks RT experience	12 weeks	1RM Bench press	Yes	3 × 3—10 Lower body 3 × 5—10 upper body (muscle failure)	1	0.98 (kg.kg^−1^)	﻿26.0 ± 3.8
								3	0.75 (kg.kg^−1^)	﻿23.8 ± 5.4
Saric et al. [30]	Resistance training frequencies 3- and 6-times per week produce similar muscular adaptations in resistance-trained men	n = 27	RT training minimum of 2 × a week for 6 months	6 weeks	1RM Bench press	Yes	4 × 6-12RM (Muscle failure)	3	1.05 (kg.kg^−1^)	﻿22.6 ± 2.1
								6		
Schoenfeld et al. [29]	Influence of resistance training frequency on muscle adaptations in well-trained men	n = 20	RT training minimum of 3 × a week for 1 year	8 weeks	1RM Bench press	Yes	2–3 × 8–12 (Muscle failure)	1 (Split)	1.19 (kg.kg^−1^)	﻿23.5 ± 2.9
								3 (Total)		
Yue et al. [36]	Comparison of two equated resistance training weekly volume routines using different frequencies on body composition and performance in trained males	n = 18	RT Experience 3.0 ± 0.5 years	6 weeks	1RM Bench press	Yes	4 × 8–12	2	0.91 (kg.kg^−1^)	﻿﻿28 ± 7.9
								4	0.97 (kg.kg^−1^)	21 ± 3.2
Zaroni et al. [37]	High resistance-training frequency enhances muscle thickness in resistance-trained men	n = 18	RT experience range from 2–10 years	8 weeks	1RM Bench press	Yes	3 × 10–12	1 (Split)	1.10 (kg.kg^−1^)	﻿26.4 ± 4.6
								5 (Total)		

*RT* resistance training, *RM* repetition maximum, *BW* bodyweight, *DOMS* delayed onset muscle soreness, *MVIC* maximum voluntary isometric contraction

### Intervention Exercise Prescriptions

Pre- to post-intervention showed trivial-to-moderate changes in maximum strength of both the lower and upper body with the majority of the interventions included within this review demonstrating the positive effects of resistance training on strength adaptations. It is, however, important to understand the potential mechanisms responsible when considering adaptations in strength. An increase in strength but no increase in muscle mass may suggest adaptations occurred predominantly due to increased fascicle length, reduction in pennation angle [38] and neural adaptations [39]. Alternatively, an increase in strength and increase in muscle mass will likely lean towards increased muscle thickness and pennation angle as well as possible increases in fascicle length [38]. This intuitively suggests that some strength adaptations will occur during hypertrophy training in response to an increase in muscle mass but may not be elevated to the level that would occur in response to a solely strength-focused training program. A summary of the studies analysed within this review can be seen in Table 2, whereby the set and repetition ranges of each of the interventions can be observed. It is clear that based upon our earlier definition of training methods seen in Sect. 1, the majority of these RT interventions are not focused on strength but heavily biased towards hypertrophy training with almost all interventions outlining sets above the recommended 3–6 repetitions [7], and most commonly employing 8–12 repetitions (see Table 2).

Despite all the interventions including exercises to RM, only three were explicitly reported to include the performance of sets to muscle failure [28, 30, 40]. The RM approach to load prescription is based upon performing the sets and repetitions with the maximum load possible to complete the full prescription, likely resulting in training to muscle failure. It is worth noting that within this review, ‘load’ is referred to when describing the amount of weight lifted, as Steele [41] and Arent et al. [42] have outlined how intensity (often used interchangeably with load) can be a better representative of effort. The reason for clearly defining the difference between load and intensity (effort) is to highlight that although the RM approach is performed with the maximum load possible for the sets and repetitions prescribed, this load may still be low–moderate, even when performed to failure and perceived to be high intensity by the athlete. Constantly training to muscle failure has been reported to have a potentially deleterious effect on performance [43]. Evidence of this effect has been observed when a group performing sets at a load relative to their maximum, compared to RM sets, demonstrated greater increases in jump performance, rapid isometric force production and muscular adaptations [43, 44]. The differences observed between the two groups is likely due to better fatigue management and potentially optimal performance adaptations, which is likely more appropriate for well-trained and professional athletes. The magnitude of load participants experienced throughout the majority of the interventions could explain why only trivial to moderate improvements in strength were observed across the 6–12 weeks. Schoenfeld et al. [40] have demonstrated similar hypertrophic responses are elicited when comparing a moderate load (three sets of 10RM) vs high load (seven sets of 3RM) when volumes are equated; however, the high load group demonstrated the greatest improvements in both back squat and bench press 1RM. In addition, well-trained populations will likely see less strength adaptations in response to hypertrophy training and specific strength training due to already having a greater base level of strength [20, 45]. Two out of the ten studies included within this review do not appear to be volume equated (Tables 2 and 3) [31, 32]. In a recent meta-analysis, Grgic et al. [21] used equated volume as a moderator, suggesting that increases in strength associated with higher frequencies of RT are largely attributed to the additional training volume. Due to the groups in studies by Hoffman et al. [32] and Kilen et al. [23] not being volume equated, it is not possible to determine whether training volumes observed in the higher frequency groups affected the resultant adaptations. The beneficial effect of increased RT volume on hypertrophic responses has previously been demonstrated [46]; however, the effect on strength is not as clear, or at which point increased volume may reduce the adaptive responses.

**Fig. 4:**
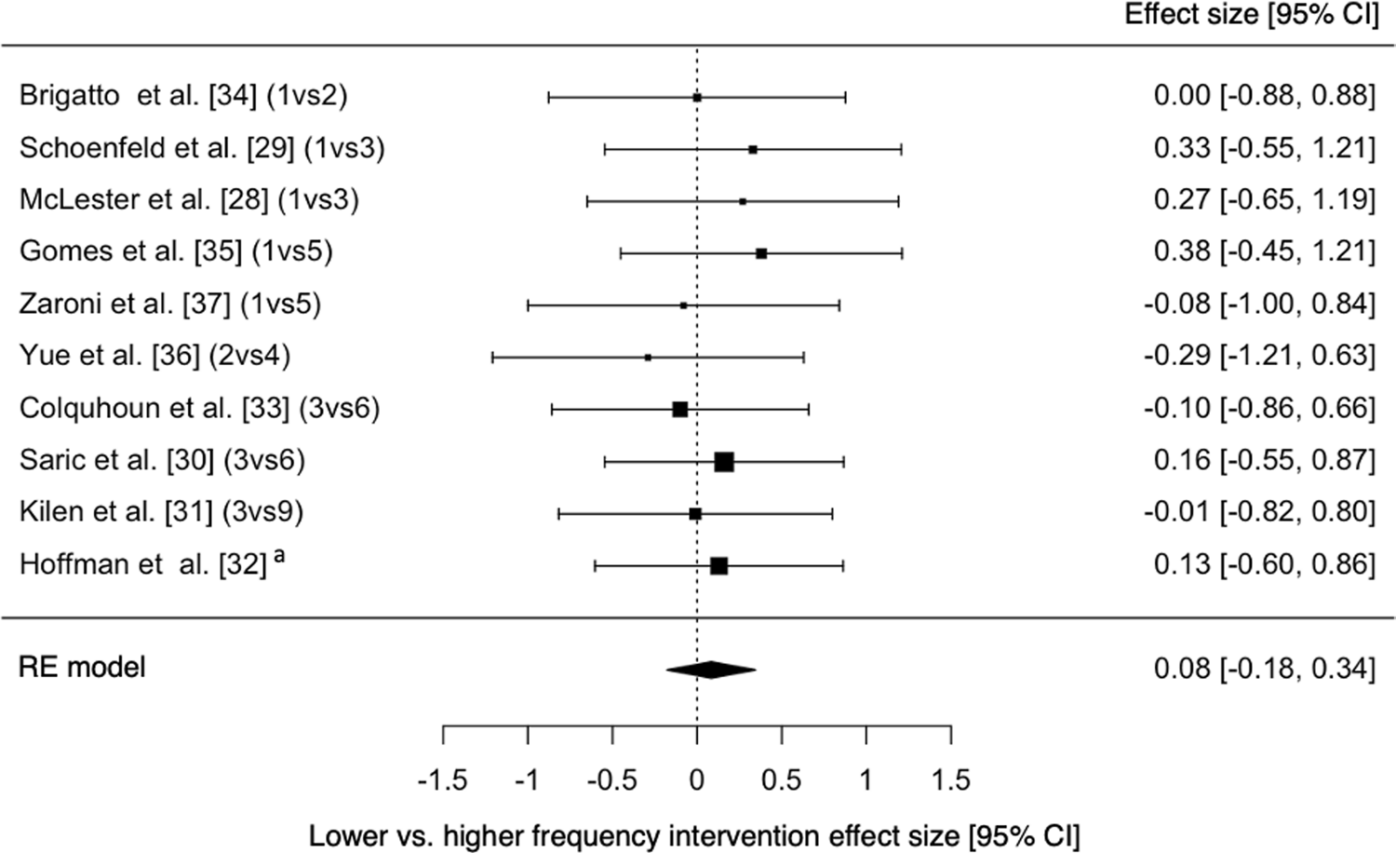
Differences in effect size between lower frequency and higher frequency groups on upper body strength (positive values favor the higher frequency groups and negative values favour the lower frequency groups). (1vs2) = once-weekly vs twice-weekly, (1vs3) = once-weekly vs 3 x/week, (1vs5) = once-weekly vs 5 x/week, (2vs4) = twice-weekly vs 4 x/week, (3vs6) = 3 x/week vs 6 x/week, (3vs9) = 3 x/week vs 9 x/week. ^a^Aggregation of effect sizes due to the study comparing more than two groups. RE = random effects, CI = confidence interval

Only one study reported negative effects in response to a lower frequency RT intervention, where the authors observed small decreases in upper body and lower body strength [31]. A potential cause for these findings could be the testing battery used. Rather than using 1RM testing, a maximum voluntary isometric contraction was used to assess the knee and elbow flexors, which was unlike the actions used within the studies training intervention. Another reason for the reduction in strength could have been due to this being the only study to use a concurrent training approach. Due to the population used (i.e., military personnel), there was a requirement to not only train muscular strength but also aerobic and muscular endurance. The requirement to train concurrently is also present in team sports, however, and the findings from Kilen et al. [31] support the complexity of this process. Due to the demands of team sports, ensuring appropriate development of all physical attributes (i.e. muscular strength and power, muscular endurance and aerobic endurance) is essential, not only to enable a greater ability to recover between efforts in training and competition but also to recover between fixtures within congested periods of a season, or during tournaments as highlighted in Sect. 1. Wilson et al. [47] have suggested that significant decrements in maximal strength may not occur as a result of endurance training but only through the incorrect training modality and/or dose. Kilen et al. [31] demonstrated the effect of traditional concurrent training whereby their “classical training” (lower frequency) group who performed training sessions of high-intensity cardiovascular, muscular endurance and strength training within their program, experienced a decrease in maximal strength. In contrast, increases in maximal strength were observed in the “micro-training” (higher frequency) group who performed the same exercises, intensity and volume, albeit divided over shorter, higher frequency bouts (Figs. 3 and 4).

### Baseline Strength Level

One of the aims of this review was to identify if RT frequency influences strength in well-trained athletes. Quantifying training experience and categorizing an athlete as ‘well-trained’ is not simple. Rhea [48] has proposed possible thresholds for *g* values to use based upon training experience (categorized as ‘untrained’, ‘recreationally trained’ and ‘highly trained’) when inspecting treatment effects. The criteria for this review, however, were for studies to state their population as well trained, but as Tables 2 and 3 outline, the variation in criteria for this population was large, ranging from 6 months to 10 years. The duration an athlete has trained for does not necessarily dictate how well trained they are, as the training they could have experienced at times throughout their career may be suboptimal. It could, therefore, be more applicable to categorize athletes based on their relative strength levels as evident within the study by Colquhoun et al. [33] who accepted subjects based upon criteria that included both length of training history and a minimum strength level (150% of bodyweight for a deadlift), similar to the selection criteria for ‘previously weight trained’ individuals outlined by Willoughby [49], of a parallel back squat 1RM ≥ 1.5 times bodyweight as this is more likely to dictate the response to the RT interventions. Relative strength levels pre-intervention have been calculated and are outlined in Tables 2 and 3. A possible reason for there being small-to-moderate changes overall regardless of frequency could be due to the populations of these studies actually being well trained as the majority of groups exceed the 1.5 times bodyweight threshold previously described for lower body strength by Willoughby [49]. The greatest difference observed between two frequencies in the lower body was observed by Yue et al. [36] (Fig. 3), an explanation for this potentially being due to the lower frequency group being the weakest at baseline in comparison to the higher frequency group and in comparison to the other studies investigated within this review. The lower relative strength results in a greater potential for improvement over the same period when exposed to the same volumes. The length of the interventions within this review could have also had an effect on the small-to-moderate change observed overall in Supplementary Information Figures S1 and S2. Unfortunately, the authors were unable to calculate relative strength changes due to a lack of reporting bodyweights post-intervention, or bodyweights for the different frequency groups rather than the whole sample population. The duration of the RT interventions included within this review was 6–12 weeks. If the athletes were well trained, as described, it is unlikely that large changes to relative strength will be observed pre-post.

### Study Quality and Bias

A Cochrane risk of bias assessment (Fig. 2) was carried out to understand the bias across the studies that used a randomized approach. The overall conclusion would be that the risk of bias is low or even slightly unclear due to lack of detail around the way the randomization was carried out six out of the eight randomized studies. The concealment of allocation was also unclear in seven out of the eight studies, with the eighth explicitly outlining that concealment of allocation did not occur. Depending upon the setting of these studies, however, that is not always ecologically possible, particularly when working in a team sport setting. Ecological validity could also provide a rationale for the lack of control groups within all but one of the studies. Given links between strength training and reduction of injuries, it could be viewed as unethical to have a control group that only takes part in the sport if they already have a background in RT as this could put them at a greater risk of injury and possible reduction in competitive advantage over those without such a background.

### Limitations and Areas for Future Research

The inclusion/exclusion criteria were initially designed to allow for a range of different potential moderators to be applied within the current meta-analysis. There was, however, a lack of consistent moderators available, which not only highlights a limitation of this review but also highlights gaps in the current literature and provides a strong rationale for future research areas in exercise prescription. The low consistency of effect (high heterogeneity) between the studies assessed in this review may have been attributable to certain commonalities. This low inconsistency is not necessarily a limitation but does highlight areas researchers need to expand on in the future. For example, all the interventions included in this review were completed on male subjects, with the vast majority being ‘recreationally trained’ and completing the same test for maximal strength. Some areas for future research would, therefore, be to investigate both sexes, but particularly females, to provide comparison with the current literature. Taking samples from athletes within different team sport settings would also be appropriate, as one set of sporting demands or type of sporting schedule may benefit from one particular approach compared to another. Research conducted within competitive team sports would, however, require the acceptance of ecological validity, whereby a number of factors that are likely outside of any investigator’s control would need to be considered. It is also important to understand that the description of team sport athletes as being ‘well-trained’ may only apply to their sport and not when to resistance training. The majority of interventions in this review included the exercises that were used to test their participants’ maximum strength (1RM). Utilizing the exercises tested within the intervention may have resulted in improvements purely based on improvement of technique or familiarization; however, due to the ‘well-trained’ nature of the population, this is unlikely. Another potential issue with the maximal testing used to assess strength was that only bench press was used as a measure of compound upper body strength, whilst most interventions included a full body approach, meaning there was a lack of evidence to demonstrate upper body strength increase as a whole. A possible limitation of only measuring maximal strength means that the rate at which the participants could produce force was not measured. The force production capabilities of athletes are important for performance and associated with injury risk reduction; therefore, not only is it important to apply force maximally, but the rate at which it is applied is also important. Measures of multi-joint rapid force production (e.g. using the isometric mid-thigh pull) should also be assessed when considering the implications for athletic populations.

Finally, as mentioned when considering concurrent training, Kilen et al. [31] described their higher frequency RT group as a “micro-training” group, and considering the overall lack of difference between training frequencies further investigation should investigate a variation of the term used by Kilen et al. [31] which has become more commonly used by practitioners which is “micro-dosing”. Micro-dosing was initially coined from a performance perspective by Hansen [50] but has not been widely used within the peer-reviewed literature, and therefore has no clear definition. We, therefore, define micro-dosing training as “the division of total volume within a micro-cycle, across frequent, short duration, repeated bouts” and suggest that such an approach should be thoroughly investigated in the future.

## Conclusion

It is evident that within the studies included in this review, there is no clear difference between RT frequencies in populations described as well trained over a 6- to 12-week period. Not knowing which method is superior may appear negative to some practitioners who are looking for clear guidance on the most efficient way to train their athletes. No clear difference between different RT frequencies is potentially a positive when trying to address the issues stated in this review around in-season training, fixture congestion and tournament schedules. The lack of difference, in agreement with previous frequency reviews, suggests that volume and load dictate adaptations in strength over frequency, which may provide the opportunity for a micro-dosing approach, meaning more frequent but shorter duration, less fatiguing bouts of RT activity, or micro-dosing. Alternatively, a more traditional approach to training may also be appropriate at times throughout a season based on the level of time constraint placed on the practitioner, providing both the volume and load are comparable between the two approaches. Researchers should look to initially assessing the effect of different RT frequencies on a strength-focused training program which uses the strength thresholds recommended by the NSCA before then exploring its interaction with pitch-based training and the possible benefits on concurrent training.

## Supplementary Information

Below is the link to the electronic supplementary material.

Supplementary file 1 (PNG 238 KB)

Supplementary file 2 (PNG 235 KB)

Supplementary file 3 (PNG 141 KB)

Supplementary file 4 (PNG 141 KB)

## Declarations

### Data Availability Statement

The data within this study are secondary data and available through the relevant articles referenced throughout. All statistical analysis was carried out using Jamovi [27] an open source software that is freely available.
