# Supplementary figures and images for: Effects of Variations in Resistance Training Frequency on Strength Development in Well-Trained Populations and Implications for In-Season Athlete Training: A Systematic Review and Meta-analysis
Source: Sports Med. 2021 Apr 22;51(9):1967–82. doi: 10.1007/s40279-021-01460-7 (PMC8363540; doi:10.1007/s40279-021-01460-7)

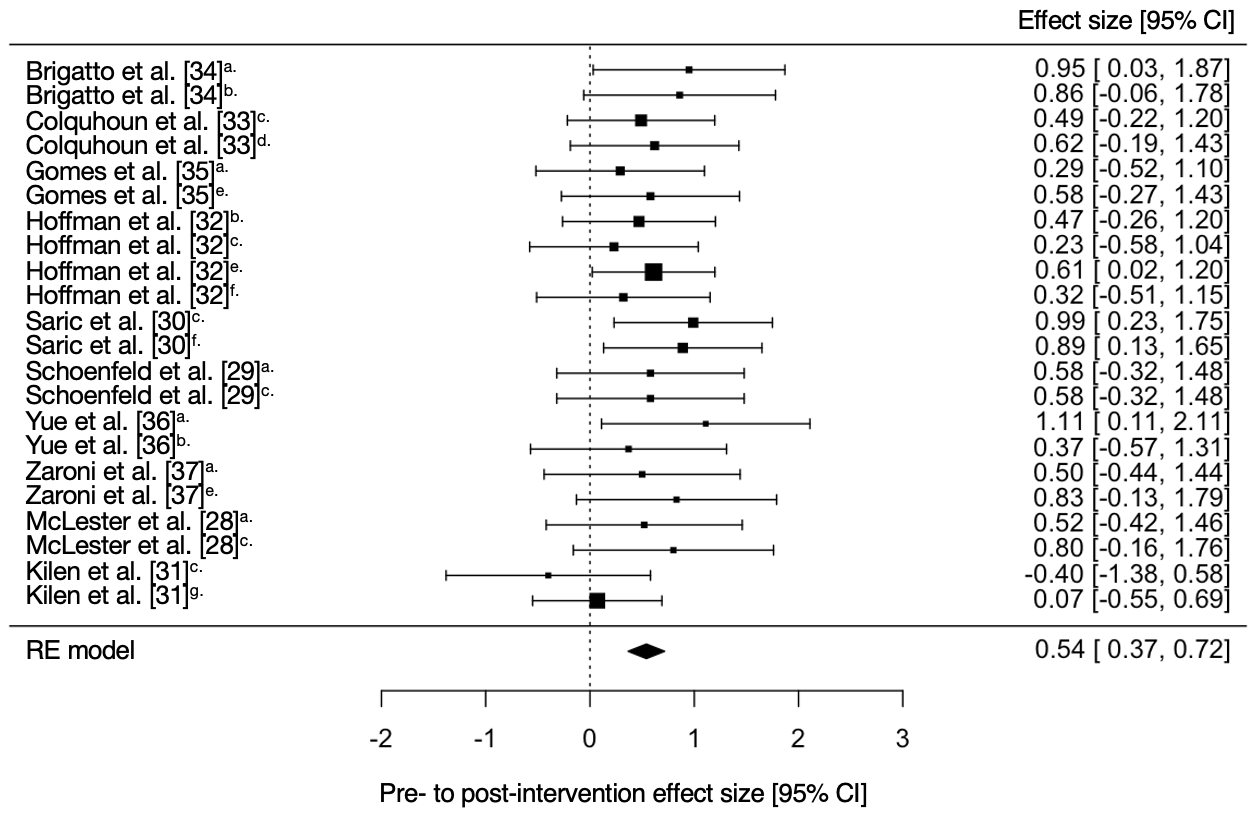

Supplement: Supplementary file 1 — (PNG 238 KB) [file 40279_2021_1460_MOESM1_ESM.png]

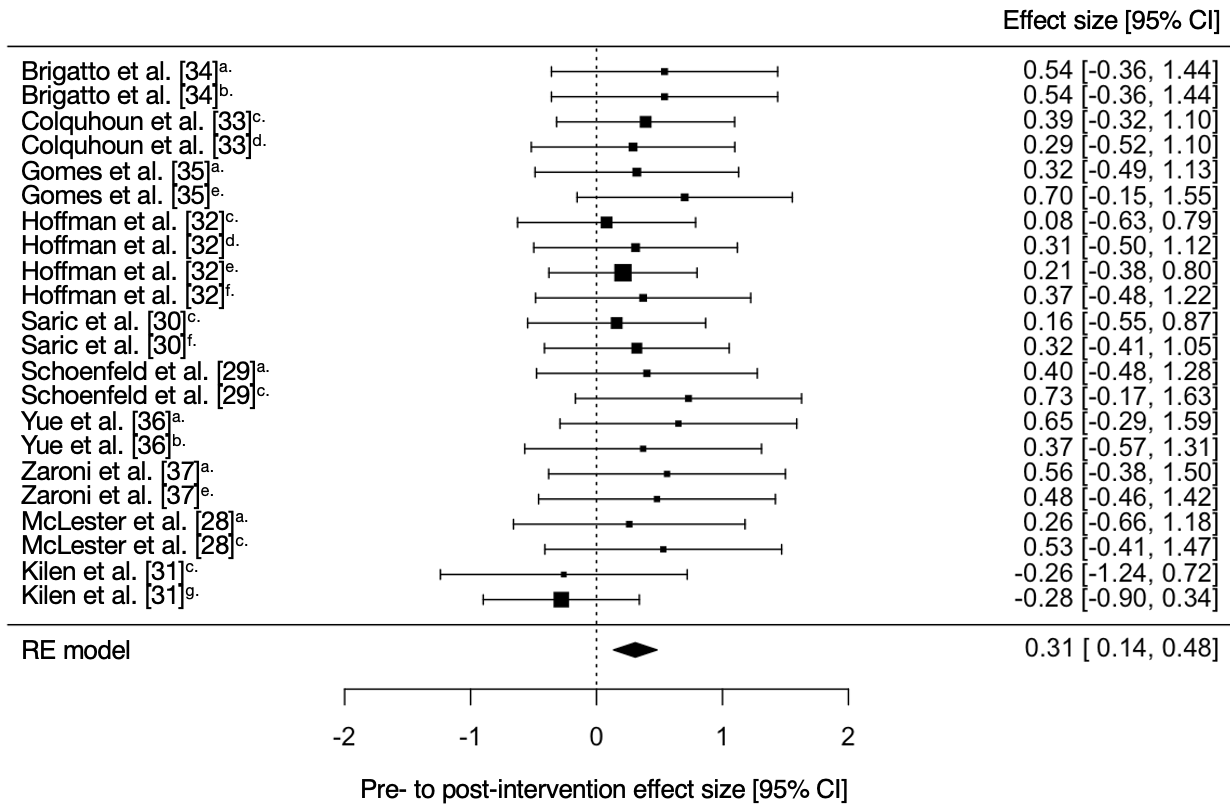

Supplement: Supplementary file 2 — (PNG 235 KB) [file 40279_2021_1460_MOESM2_ESM.png]

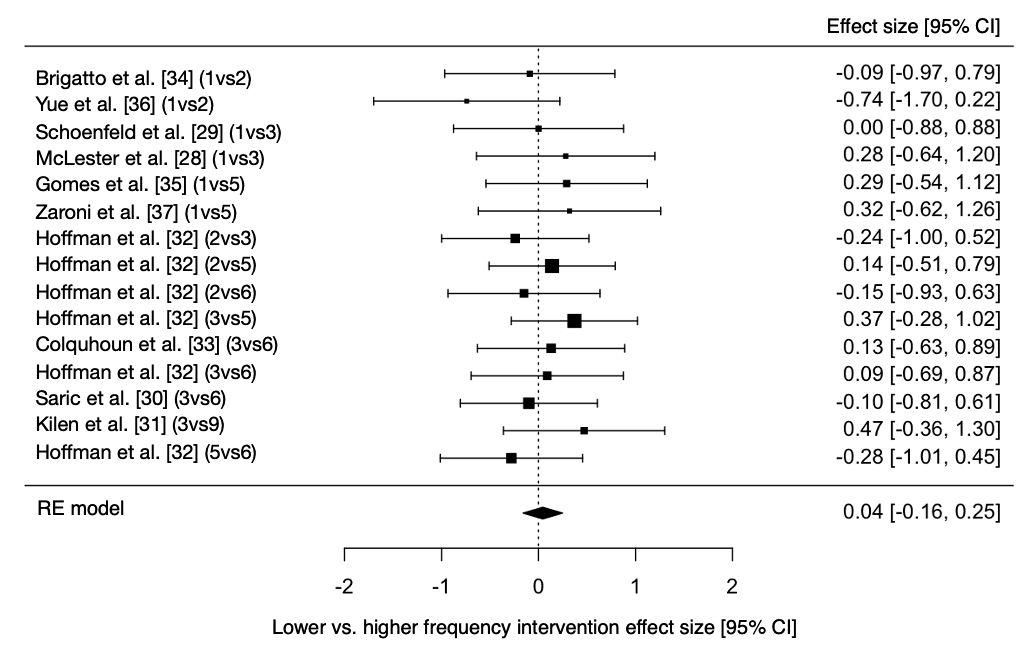

Supplement: Supplementary file 3 — (PNG 141 KB) [file 40279_2021_1460_MOESM3_ESM.png]
